# Characterization of an orthotopic gastric cancer mouse model with lymph node and organ metastases using bioluminescence imaging

**DOI:** 10.3892/ol.2020.11860

**Published:** 2020-07-10

**Authors:** Hai-Yi Feng, Yunpeng Zhang, Hai-Jun Liu, Xiao Dong, Si-Cong Yang, Qin Lu, Fanping Meng, Hong-Zhuan Chen, Peng Sun, Chao Fang

Oncol Lett 16: 5179-5185, 2018; DOI: 10.3892/ol.2018.9313

Subsequently to the publication of the above article, the authors have realized that a panel was selected incorrectly in Fig. 5A. Essentially, the heart photo of mouse #2 at week 6 was inadvertently misplaced (the image shown was a duplication of the image for mouse #1 at week 6).

A corrected version of Fig. 5, including the correct data for the heart photo of mouse #2 at week 6, is shown opposite (an enlargement of the data for mouse #2 specifically for week 6 is also shown on the next page as Fig. S1 for the perusal of the readers). Note that this change does not affect the results or the conclusions reported in this paper, and all the authors agree to this correction. The authors thank the Editor for allowing them the opportunity to publish this corrigendum, and apologize to the readership of the Journal for any inconvenience caused.

## Figures and Tables

**Figure 5.**
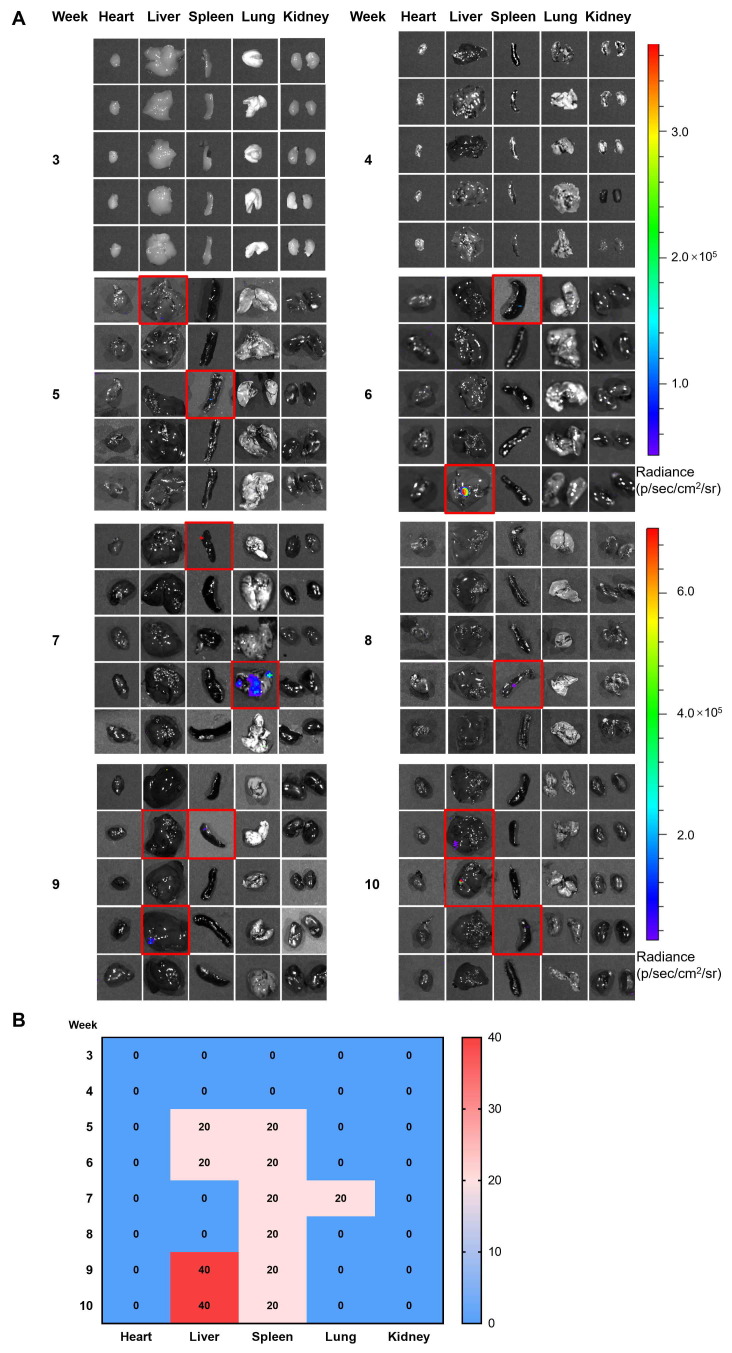
The metastasis in major organs of the gastric cancer mice model. From week 3–10, mice (n=5 for each week) were sacrificed and the major organs, including the heart, liver, spleen, lung and kidney, were resected for metastasis detection. Note that the images presented for each week are not from the same mouse. (A) The metastases were detected using the bioluminescence imaging. The red rectangles indicated the identified metastatic organs. Each row represents one mouse. (B) The time-dependent metastasis frequencies in the major organs were summarized in the heat map.

**Figure S1.**
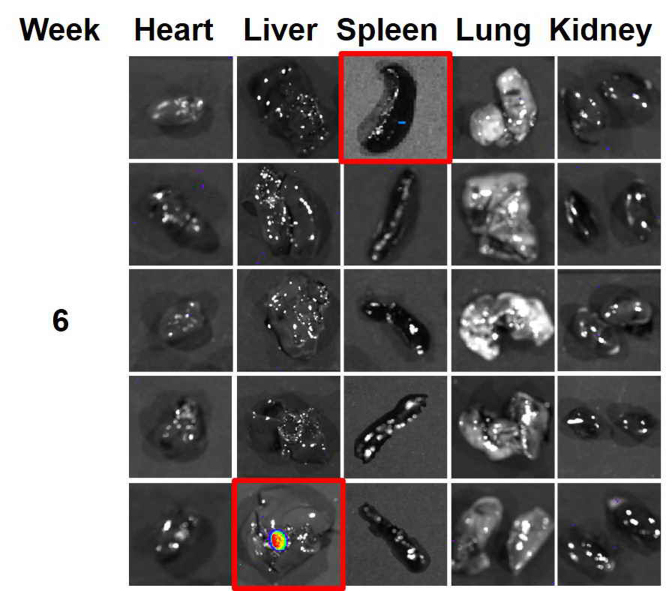
An enlarged version of the heart photo shown in Fig. 5A for mouse #2 for week 6 is included here, for the purposes of highlighting the data.

